# The effects of progressive muscular relaxation as a nursing procedure used for those who suffer from stress due to multiple sclerosis**^1^**

**DOI:** 10.1590/1518-8345.1257.2789

**Published:** 2016-09-01

**Authors:** Paolla Gabrielle Nascimento Novais, Karla de Melo Batista, Eliane da Silva Grazziano, Maria Helena Costa Amorim

**Affiliations:** 2 RN, Departamento de Educação Integrada em Saúde, Universidade Federal do Espírito Santo, Vitória, ES, Brazil; 3 PhD, Adjunct Professor, Departamento de Enfermagem, Universidade Federal do Espírito Snto, Vitória, ES, Brazil.; 4 PhD, Adjunct Professor, Departamento de Enfermagem, Universidade Federal de São Carlos, São Carlos, SP, Brazil.; 5 PhD, Associate Professor, Departamento de Enfermagem, Universidade Federal do Espírito Santo, Vitória, ES, Brazil.

**Keywords:** Nursing, Relaxation, Psychological Stress, Multiple Sclerosis

## Abstract

**Objective:**

to evaluate the effect of progressive muscle relaxation as a nursing procedure on the levels of stress for sufferers of multiple sclerosis.

**Method:**

random clinical trials conducted at the Neurology outpatients unit at a University Hospital. The sample consisted of 40 patients who were being monitored as outpatients (20 in a control group and 20 in an experimental group). The Progressive Muscle Relaxation technique was used. The control variables were collected through interviews that were recorded on forms and on the Perceived Stress Scale that we used. Five meetings were held every fortnight covering a period of eight weeks. The experimental group was advised to carry out daily progressive muscle relaxation activities. After eight weeks of these activities, they were evaluated again to measure their levels of stress. In order to analyze the data used, the software package Statistics for Social Sciences version 19.0 was used.

**Results:**

the application of the t test showed a significant reduction in the Perceived Stress Scale scores in the experimental group (p<0.001), which in turn proved that there was a reduction in the levels of stress after the application of the relaxation practic-es.

**Conclusion:**

the progressive muscle relaxation activities contributed to the reduction in stress levels for multiple sclerosis suffers and thus can be used in nursing for patients. Clinical Trials Identifier: NCT 02673827.

## Introduction

Multiple Sclerosis (MS) is a chronic, autoimmune disease which is characterized by neuro-degeneration of the central nervous system which presents a number of consequences such as: variable motor deficits and sensitivities caused by the demyelination of the myelin sheath. It affects young adults in the prime of their productive lives bringing about permanent neurological incapacity in the long term^1^. It is also associated with: psychological alterations, cognitive deficits, fatigue, emotional problems and stress which affects their ^2^ quality of life. 

Research has proven an association between stress and the worse clinical cases of MS. Stress can influence the onset of MS and its clinical development making it more intense and increasing the frequency of the symptoms ^3^ ^-^ ^4^. Information has also shown that there are negative associations between stress and the quality of life for those with MS^5^ ^-^ ^6^.

MS is a complex, multifactorial condition of unknown etiology covering genetic and environmental factors. The neuroendocrine system can be influenced by intrinsic and extrinsic elements such as emotional and psychological stress. The immune and neuroendocrine systems communicate with each other in a bidirectional way through receptors and common molecular messengers. Any type of level alterations can bring about susceptibility in changes and the levels of gravity in autoimmune and inflammatory diseases such as MS^7^.

Negative stressful events and psychological suffering worsen neurological symptoms and increase the risks of additional cerebral lesions detected through MRI scans on patients with MS^8^_._ Known information relates emotional stress with an exacerbation of neurological symptoms. This is due to inflammatory factors induced by stress which damages the conductance of the demyelinated axons or somatization disorders where neuro-mechanisms are affected^3^.

However tackling stress can be even more important for those incapacitated by stress such as MS suffers in relation to the population as a whole. This is because these people not only experience more stress but they can also be more vulnerable to its negative effects^9^.

Amongst the varies option that exist for tackling stress, relaxation techniques are being used on a more frequent basis in order to relieve every day stress. It is considered one of the more simple and easy to use methods to put into practice ^10^. Acute stress constitutes the biggest impact in relation to exacerbating the disease with reference to chronic stress in patients with MS. This highlights the importance of tacking an approach where patients are instructed on the techniques to be used to tackle stress ^11^.

The Progressive Muscle Relaxation method (RMP) is an active, participative and dynamic method that promotes the independence of the individual. It allows people to learn to assess their specific muscular group tensions in order to relax them ^12^.

Recent research on the effects of RMP on patients with MS has covered interventions made on suffers of: fatigue, poor quality of sleep, stress and depression. All of these studies presented scientific evidence that RMP brought a better quality of life for the patients ^13^ ^-^ ^15^.

In Brazil the legislation covers the nursing profession and the implementation of alternative treatments with the aim of promoting patients health through natural, traditional, complementary and non-conventional therapies ^16^. 

In the face of scientific evidence and the scarcity of national studies on the use of RMP as a therapeutic intervention in nursing for suffers of MS, the following question emerged: what is the effect of RMP on stress levels for suffers of MS considering the theory that RMP reduces stress levels for those with the condition? With the aforementioned in mind, the objective of this study was to evaluate the effects of RMP on patients' stress levels.

## Method

Random clinical trials were undertaken. 40 patients with MS took part in this research who were being monitored as outpatients at the Cassiano Antônio de Moraes Neurology University Hospital which is a part of the Espírito Santo Federal University. They were chosen due to meeting the following inclusion criteria: they had been diagnosed with relapsing-remitting MS for about six months, they had been undergoing immunotherapy, they registered ≤ 5.0 on the Expanded Disability Status Scale (EDSS), they were aged between 18 and 65 years old, they had not had any relapses in the three months that ran up to the start of the study and they were living in the metropolitan region of Vitória-ES. The exclusion criteria was: being hospitalized or having a relapse when the data was being collected, showing physical or mental problems that would prevent the collection of data, having motor or cognitive deficits, constantly using psychotropic medication (for example antidepressants, benzodiazepines, antipsychotic drugs or other stimulants) or using integrative practices and complementary health therapies (for example, yoga, pilates, meditation, psychotherapy).

The outpatients unit at the Neurology center is a center of excellence in the treatment of MS in the state of Espírito Santo attending patients from other states such as Minas Gerais and Bahia totaling about 300 patients. After an analysis of the inclusion and exclusion criteria, 60 individuals were chosen. In order to calculate the sample number from the 60 individuals, we factored in a 5% sample error and a confidence level of 90% which produced a sample of 50 individuals. All of the 60 patients were invited to take part in the research as we foresaw loses. However: 9 refuses to take part, 5 changed their addresses, 3 had their diagnosis changed, 2 had relapses and 1 was pregnant. Out of the aforementioned people, the study finally settled for 40 people that had been diagnosed as having MS and who were being monitored as outpatients in the aforementioned hospital. They had been randomly selected using a computer program that generates numbers at the site *www.randomizer.org* *.* This randomization occurs after having typed in information on sex, age and diagnosis for the control group (n=20) and the experimental group (n=20). 

The variable information such as: sex, age, marital status, race/color, level of education, occupation, time of diagnosis and medication to control the condition was collected through interviews and filling out our research specific forms. 

Five meetings took place with intervals of 15 day between them. In order to avoid bias, a Field Diary was used by researchers covering the daily activities of the participants. This information was obtained from the fortnightly meetings and weekly telephone calls.

In order to avoid the *Hawthorne* effect where there is contamination between the control group and the experimental group, we took care in not scheduling participants' meetings at the same times, so that they would not meet each other. In relation to the meetings:

-First Meeting: Individual meetings with the patients from the experimental and control group were carried out at the outpatients unit at the Neurology center after appointments had been made. For both of the groups, through interviews, a socio-demographic form and the Perceived Stress Scale were used (PSS- 10). This scale is made up of 10 questions that were the *Likert* type that varied from zero meaning "never" to 4 meaning "almost always". Question 4, 5, 7 and 8 had reverse points, inverting the values shown on the scale. A determination of stress levels is obtained through the sum of all of the items that comply with the aforementioned inverted point system, meaning a high points score constituted high levels of stress^17^. 

The experimental group was required to carry out the Progressive Muscle Relaxation technique (RMP) using an audio CD and explanation leaflet produced for this study describing the steps to be followed. They were told to practice RMP once per day during eight weeks in their households during the day when they were not that tired.

To evaluate whether the participant obtained a relaxed state, some physiological parameters were captured and measured before and after the RMP intervention. Therefore we measured their blood pressure (PA) and heart frequency (FC) using a digital automatic monitor known as the OMRON 705 CP. Measurements of respiratory frequency (FR) was done through observing the diaphragmatic respiration for a period of a minute using a conventional watch. It should be noted that for measuring physiological parameters, there were no interferences or interruptions in the relaxation.

-Second, Third and Fourth meetings: For both groups, we carried out three meetings with intervals of 15 days in the Neurology outpatient units. During the meetings for both groups, an appointment with a nurse took place, with the objective of evaluating and monitoring the development of MS. 

The participants of the experimental group also executed the technique under the supervision of a researcher and they submitted themselves to measuring their PA, FC and FR. They were contacted once per week via a telephone call to evaluate their state of health. For the experimental group, this contact allowed for the next meeting to be scheduled as well as to check that the RMP was being correctly done. This time was also used to encourage them to continue.

 In the intervals between the three meetings for the participants in the experimental group, they were visited by researchers at their households in order to evaluate the carrying out of the RMP technique and to measure their physiological parameters: blood pressure (PA) and heart frequency (FC). Both were measured using the OMRON 705 CP digital automatic monitor. Respiratory frequency (FR) was also measured through observing diaphragmatic respiration during a period of one minute using a conventional watch. The physiological parameters were used as physiological markers for RMP.

-Last meeting: After eight weeks from the start of the intervention, the participants (experimental group and control group) returned to the Neurology center's outpatients unit for a last check up with a nurse. For the experimental group, we carried out the RMP on them and then we measured their FC, FR and PA before and after the intervention. We used the PSS-10 on both groups.

In order to analyze the statistical data we used the Statistical Package for Social Sciences (SPSS) version 19.0 with a 5% level of confidence corresponding to p=0.05 (confidence limit at 95%). We initially noted through the *Shapiro-Wilk* test of normality that the captured data had a normal distribution (GAUSS). Nevertheless we applied the *t* Test for averages which is a parametric test for independent samples geared at the comparison of two groups when there are no breaks in the normality theory. We used the non-parametric *Wilcoxon* Test in relation to comparisons between moments in each group. 

This research was approved by the Ethics Committee for Research at the Science Center for Health at the Federal University of Espírito Santo under record number 618.841. It can also be found on the site ClinicalTrials.gov under number NCT02673827. 

## Results 

Based on the characteristics of the sample and the measured correlations of the experimental and control groups covering their variables (sex, age group, race/color, marital status, level of education, occupation, period of time with the condition and medication) we saw a homogeneity in the groups, that allowed them to be compared. Table 1 illustrates the characteristics of the groups. 

**Table 1:** Characteristics of the sample: absolute numbers and percentages according to groups. Vitória, ES, Brazil, 2014

Variables		Experimental			Control		p-valor
		n	%		n	%	
Sex							
	Female	15	75.0		14	70.0	1.000
	Male	5	25.0		6	30.0	1.000
Age group							
	Up to the age of 20	-	-		1	5.0	1.000
	21 to 30 years old	4	20.0		7	35.0	0.479
	31 to 40 years old	6	30.0		5	25.0	1.000
	41 to 50 years old	8	40.0		3	15.0	0.157
	51 to 60 years old	2	10.0		4	20.0	0.658
Race/Color							
	White	11	55.0		16	80.0	0.177
	Mixed Race	8	40.0		4	20.0	0.301
	Black	1	5.0		-	-	1.000
Marital status							
	Single	6	30.0		10	50.0	0.333
	Married	13	65.0		9	45.0	0.340
	Widowed	1	5.0		-	-	1.000
	Divorced	-	-		1	5.0	1.000
Level of education							
	Primary School Education Incompleted	4	20.0		1	5.0	0.339
	Primary School Education Completed	1	5.0		1	5.0	0.468
	High School Education Incompleted	-	-		1	5.0	1.000
	High School Education Completed	8	40.0		9	45.0	1.000
	University Education Incompleted	3	15.0		3	15.0	0.658
	University Education Completed	3	15.0		3	15.0	0.658
	Post Graduate Education	1	5.0		2	10.0	1.000
Occupation							
	Informal work	3	15.0		1	5.0	0.598
	Formal work under a work contract	4	20.0		8	40.0	0.301
	Public Sector Worker	-	-		1	5.0	1.000
	Retired	8	40.0		4	20.0	0.301
	Supported due to suffering from a disease	1	5.0		3	15.0	0.598
	House wife	3	15.0		1	5.0	0.598
	Student	1	5.0		2	10.0	1.000
Period of time with the diagnosed condition							
	1 to 5	8	40.0		12	60.0	0.343
	6 to 10	8	40.0		3	15.0	0.157
	11 to 15	3	15.0		3	15.0	0.658
	16 to 20	-	-		1	5.0	1.000
	Above 20	1	5.0		1	5.0	0.468
Controlled Medication							
	Natalizumabe	7	35.0		5	25.0	0.730
	Beta Interferon	3	15.0		9	45.0	0.085
	Rebife	2	10.0		3	15.0	1.000
	Gylenia	7	35.0		2	10.0	0.130
	Avonex	1	5.0		1	5.0	0.468
Total		20	100.0		20	100.0	-

*The z test for proportions. p-value < 0.050

In relation to the analysis of stress levels, the study used the *Perceived Stress Scale* (PSS-10), which showed a *Cronbach* alph of 0.756 (showing internal consistency and homogeneity between the items). 

**Figure 1:**
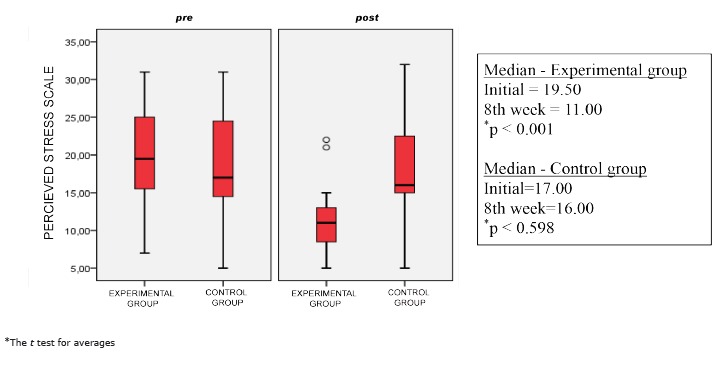
Comparisons of the stress levels for people with MS, experimental and control groups, at the start and after eight weeks of relaxation intervention, Vitória, ES, Brazil, 2014

Figure 1 shows that the experimental and control groups presented, in the first moments, similar stress levels with medians being at 19.60 and 17.00 respectively. The averages were 20.45 and 18, as well as 65 and moving from the norm of 6.63 and 6.38 and a p-value of 0.387. All showed that the groups were homogeneous. 

After eight weeks of intervention the median of the PSS-10 in the experimental group was 11.00 and for the control group it was 16.00. We noted a small median reduction of the PSS-10 for the control group but it was not significant (p=0.598), whilst there was a significant statistical reduction in the experimental group (p<0.001).

The correlations between the groups were not measured (p>0.05).

For the experimental group, in all of the meetings there was a statistically significant difference (p <0.005) in relation to the stress levels and the biological markers (PA, FC and FR) in the initial moments before and after the relaxation technique were used. This came with a reduction in the physiological parameters after the intervention, showing that the RMP intervention was effective (Table 2).

**Table 2:** Descriptive statistics and results of the comparison test between the pre and post moments in every meeting (E), in relation to the heart frequency (FC), the respiratory frequency (FR), systolic blood pressure (PAS) and diastolic blood pressure (PAD) - Experimental Group. Vitória, ES, Brazil, 2014

Variable	Moment	Median	Average	Divergence from the norm	95% IC Lim.inf.	95% IC Lim.sup.	p-value
FC (E1)*	Pre	77,50	77,20	9,08	72,95	81,45	p < 0.001*
	Post	69,00	70,85	9,46	66,42	75,28	
FC (E 2)	Pre	76,50	78,45	12,57	72,57	84,33	< 0.001
	Post	74,00	74,20	11,80	68,68	79,72	
FC (E3)	Pre	78,00	78,10	11,79	72,58	83,62	< 0.001
	Post	74,50	73,95	10,27	69,14	78,76	
FC (E4)	Pre	79,00	77,20	10,57	72,25	82,15	< 0.001
	Post	74,00	72,75	9,40	68,35	77,15	
FC (E5)	Pre	73,00	74,85	10,71	69,84	79,86	< 0.001
	Post	70,50	70,75	8,56	66,74	74,76	
FR (E1)*	Pre	19,00	18,95	1,73	18,14	19,76	< 0.001
	Post	16,00	16,30	1,63	15,54	17,06	
FR (E2)	Pre	18,00	18,00	1,45	17,32	18,68	< 0.001
	Post	16,00	15,85	0,99	15,39	16,31	
FR (E 3)	Pre	18,00	18,00	1,52	17,29	18,71	< 0.001
	Post	15,50	15,45	1,23	14,87	16,03	
FR (E 4)	Pre	17,00	17,50	1,15	16,96	18,04	< 0.001
	Post	15,00	15,30	1,22	14,73	15,87	
FR (E5)	Pre	18,00	17,60	1,19	17,04	16,16	< 0.001
	Post	15,50	15,50	0,95	15,06	15,94	
PAS (E1)*	Pre	72,00	75,00	12,43	69,18	80,82	0,002
	Post	67,50	72,00	12,14	66,32	77,68	
PAS (E2)	Pre	74,00	76,75	16,74	68,92	84,58	<0,001
	Post	70,50	72,55	11,18	67,32	77,78	
PAS (E3)	Pre	77,00	78,75	12,13	73,07	84,43	<0,001
	Post	70,00	72,60	11,08	67,41	77,79	
PAS (E4)	Pre	76,50	78,80	15,81	71,40	86,20	<0,001
	Post	72,50	73,10	10,08	68,38	77,82	
PAS (E5)	Pre	77,00	74,25	10,52	69,33	79,17	<0,001
Post	71,00	71,20	9,65	66,68	75,72

*The non-parametric Wilcoxon test

## Discussion

Finding responses to stress is a dynamic and individual process. Factors such as one's personality, life experience, and both cognitive and biological factors can influence the evaluation of an individual in relation to potentially stressful events^18^. Biological responses to stress involve various systems, including the: autonomous nervous systems, the hypothalaic-pituitary-adrenal axis and the vascular system. All of these systems are intimately connected to both innate immune responses, therefore stress may affect the immune system in an immune disease such a MS^4^.

Patients with MS present signs of hyperactivity in the hypothalaic-pituitary-adrenal axis. Aside from this, the sympathetic adrenomedullary system, pro-inflammatory cytokines and mast cells can find themselves being altered^19^. However, alterations in the physiology of the stress may affect the response to stress for people with MS. 

One study evaluated the relations between the symptomatology of the disease, the perceived stress and the productions of cytokines from mononuclear cells taken from peripheral blood in 42 MS patients in outpatient units. The production of interleukin IL-6 and IL-10 correlated itself positively with psychological stress, mood swings and the symptomatology of the disease for patients with MS, when compared with the control groups. This then made it important that the individual with MS received help to develop positive strategies to tackle the condition which were effective in improving psychological stress levels and reducing the effects of the condition^20^.

Stressful situations alter the homeostasis, triggering off a sympathetic reaction in the organism which elevates the blood pressure, heart frequency and respiration amongst other factors. RMP restores the balance in the organism, acting in opposition to the alarm phase of the stress. This in turn reduces blood pressure, heart frequency and respiratory frequency due to the reduction in sympathetic activity and the increase in vagal activity^21^. PA, FC and FR are important clinical parameters in evaluating the effectiveness and quality of the proposed relaxation technique. This being the case, it is therefore necessary that in the area of nursing, care strategies are implemented to promote better action against stress which can reduce relapses or the worsening of the condition.

The results of this study show a significant reduction in stress levels after eight weeks of RMP intervention. This is proven through the significant reduction in the clinical parameters (FC, FR, and PA) after having carried out the relaxation techniques. There were also reductions in the Perceived Stress Scale scores. 

Similar results were found through the use of RMP as long as the intervention took place which brought about reductions in stress levels for MS suffers. The results showed a significant effect in the reduction of perceived stress after eight weeks of intervention in association with the Respiration technique^10^.

The use of relaxation techniques as an intervention to tackle stress for people with MS can be accompanied by other practices including cognitive-behavioral strategies. This has demonstrated long term progress in relation to tackling stress^22^. We saw similar results for disabled women including MS suffers showing reductions in stress levels after six weeks of intervention^9^.

A program of stress management therapy based on cognitive-behavioral therapy which includes relaxation techniques for an intervention period of 24 weeks, reduced the number of lesions identified by MIR scans^23^. 

A study that evaluated psychological interventions for patients with MS in which the experimental group carried out the *Biofeedback* technique with the RMP and Respiration technique and where the control group just practiced RMP and the Respiration technique, showed that improvements were made in perceived stress for both groups. It was suggested that this was the case due to, in part, the daily practice of RMP with the Respiration technique^24^.

The practice in the use of RMP by itself or in conjunction with other practices has produced satisfactory results in the reduction of stress for MS suffers. The use of the integrative practice has become an important tool for tackling this condition. This is a form of accessible technology that is non-invasive that contributes to mental and corporal balance aiding in the reduction of anguish, emotional instability and stress.

Patients with MS require systemized and humane care. It is important for there to be the implementation of strategies that promote the better adaption and reduction of the impact of stress on them. The relaxation techniques provide physical and mental balance which improves the skills for individuals to handle stressful situations considering both the condition and the needs of the people.

Stress can seriously affect the quality of life of patients with MS which contributes to their suffering the worse part of the illness. As a result it becomes essential to evaluate the stress levels of these individuals and to develop proposals to tackle stressful situations using innovative technologies (such as relaxation) which can help the patients' life.

Although this study has shown that RMP can be used as a possible intervention for stress, there were some limitations. 

The size of the sample was a limiting factor in this study because it did not allow for generalization to be made based on the results. It also did not permit statistical non-significance in the analysis to really be considered as the absence of an intervention effect. Another limiting factor was our not using laboratory examinations such as IGA and salivary cortisol that would have been important to complement the data obtained from the Perceived Stress Scale which makes it possible to conduct statistical analysis. Also the condition that the participants "reside in the metropolitan region" was a limiting factor because we were unable to replace the losses in participants.

We suggest that future studies should go beyond our preliminary conclusions using a larger sample of people and through analyzing other similar techniques. There should also be the use of laboratory exams.

## Conclusion

There was a significant reduction in stress levels for suffers with MS for the experimental group (p<0.001), after having the RMP intervention when compared with the control group (p=0.598). Based on the results that we obtained, we noted that RMP reduced stress levels in patients with MS. This is an intervention that is simple, accessible, provided at low cost and can be done during medical appoints to see a nurse. It can also be done by the patient in their own homes which, in turn, gives them greater autonomy as well as a greater connection with those from whom they are receiving medical assistance. Aside from this, RMP as an intervention that can be practiced by nurses, increases and broadens their professional capabilities both in outpatient units and when conducting house visits.
